# Free-Radical Polymer Science Structural Cancer Model: A Review

**DOI:** 10.1155/2013/143589

**Published:** 2013-03-04

**Authors:** Richard C. Petersen

**Affiliations:** Department of Biomaterials and Biomedical Engineering, The University of Alabama at Birmingham, SDB 539, 1919 7th Avenue South, Birmingham, AL 35294, USA

## Abstract

Polymer free-radical lipid alkene chain-growth biological models particularly for hypoxic cellular mitochondrial metabolic waste can be used to better understand abnormal cancer cell morphology and invasive metastasis. Without oxygen as the final electron acceptor for mitochondrial energy synthesis, protons cannot combine to form water and instead mitochondria produce free radicals and acid during hypoxia. Nonuniform bond-length shrinkage of membranes related to erratic free-radical covalent crosslinking can explain cancer-cell pleomorphism with epithelial-mesenchymal transition for irregular membrane borders that “ruffle” and warp over stiff underlying actin fibers. Further, mitochondrial hypoxic conditions produce acid that can cause molecular degradation. Subsequent low pH-activated enzymes then provide paths for invasive cell movement through tissue and eventually blood-born metastasis. Although free-radical crosslinking creates irregularly shaped membranes with structural actin-polymerized fiber extensions as filopodia and lamellipodia, due to rapid cell division the overall cell modulus (approximately stiffness) is lower than normal cells. When combined with low pH-activated enzymes and lower modulus cells, smaller cancer stem cells subsequently have a large advantage to follow molecular destructive pathways and leave the central tumor. In addition, forward structural spike-like lamellipodia protrusions can leverage to force lower-modulus cancer cells through narrow openings. By squeezing and deforming even smaller to allow for easier movement through difficult passageways, cancer cells can travel into adjacent tissues or possibly metastasize through the blood to new tissue.

## 1. Introduction 

**Figure 1:**
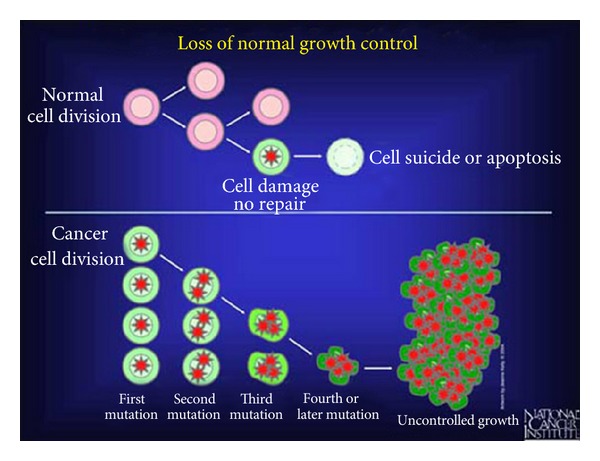
Normal cells control growth by programmed death known as apoptosis. Cancer cells conversely build genetic mutations that can result in uncontrolled growth after at least the fourth mutation. (With permission from the National Institutes of Health/Department of Health and Human Services).

*Cancer Fundamentals*. Cancer is a pathological condition related to malignant uncontrolled rapid cell growth proliferation, invasive cell movement into adjacent tissues, and occasional metastatic spread through blood and lymph to more distant locations [1–4]. Conversely, benign tumors represent uncontrolled cell growth that does not invade other tissues [1–4]. Cancers are the result of progressive accumulations in genetic mutations through cell interactions with carcinogens such as tobacco, sunlight, radiation, infectious microbes, or certain chemicals/material [1–4]. Some genetic changes can be added by being passed along from one generation to another to increase cancer risk [1–4]. Although normal cells have limits to replication or the number of cell divisions to control growth by apoptosis cell death when necessary with a cascade of caspase enzymes, cancer cells can develop almost limitless uncontrolled growth after at least four genetic mutations [1–4], Figure 1. 

**Figure 2:**
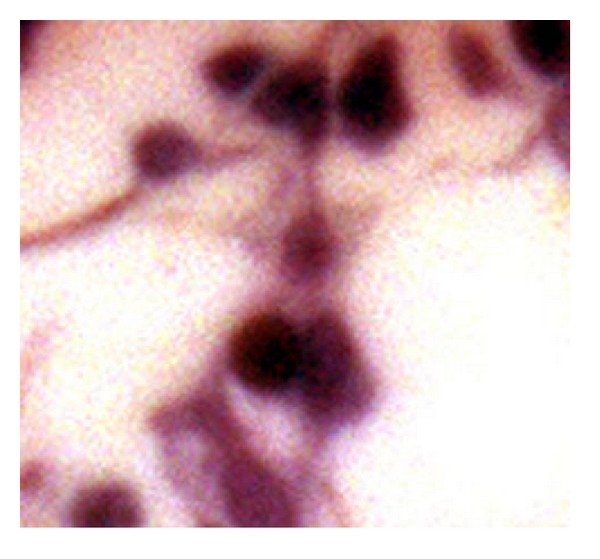
Mesenchymal stem cell with more lightly stained nucleus in bone marrow central to differentiating preosteoblastic stem cells above and below the stellate cell acquired during histomorphometry analysis.

Cancer cells can thus become less prone to death so that unneeded cells develop to form extra tissue known as tumors [1–4]. However, benign tumors that localize and do not invade adjacent tissue are not cancer [1–4]. Benign tumors are further often encapsulated by connective tissue [1, 4]. Conversely, malignant tumors invade adjacent tissues and can enter the blood stream to attack other organs by metastasis [1, 2, 4]. In order to become motile and invade into other tissues, major genetic cell traits change involving mutation through a process termed the epithelial-mesenchymal transition (EMT) [4]. Several important cellular alterations occur during EMT to include loss of integrin attachment between cytoskeleton actin fibers with adjacent cells and subsequent motility with invasiveness [4]. Further, EMT is characterized by large variations in cell shape that comprise loss of spheroid rounding with formation of spindle-shaped or fibroblast-like cells and irregular membrane borders especially at the invasive tumor edge [4]. EMT may also consist of possible long growth processes [4]. During EMT, cells dedifferentiate from the normal tissue phenotype toward the more primitive mesenchymal stem cell [4]. To better understand EMT by comparison between carcinoma cells derived from the epithelium with smooth normal cell membranes tightly bound together, a classical stellate mesenchymal stem cell with membrane extensions is isolated free in bone marrow extracellular space and surrounded by advancing differentiated preosteoblastic stem cells, Figure 2.

A more complete list of cancer causing risk factors includes the following.

1. Age increases cancer risk probably by additive effects of genetic mutation and exposures [1, 3, 4].
2. Tobacco is the most widely known cause of death and directly related to an extremely high level of cancer mortality. About 20–30 percent of all cancer deaths are related to tobacco. Cancers that increase in risk with smoking are found in the lung (highest percent of cancer-related deaths), mouth, throat, larynx, esophagus, stomach, pancreas, bladder, kidney, cervix, and in the blood with myeloid leukemia [1–5].
3. Ultraviolet (UV) light from the sun or other sources for tanning can initiate aging and eventually cancer [1, 3, 4, 6].
4. Ionizing radiation from radioactive sources such as nuclear explosions increases cancer for leukemia, the thyroid, breast, lung and stomach, and radon gas formed in the earth to increase lung cancer. Other sources of radiation include X-rays and cosmic rays from space [1, 3, 4, 7].
5. Chemicals and materials from a multitude of various sources can increase cancer risk from arsenic, asbestos, styrene, benzene, benzidine, cadmium, nickel, or vinyl chloride. Carcinogenesis by chemicals was shown to occur by an initiation step with a primary application and then an irritation step that promotes cancer [1, 3, 4, 8].
6. Infectious microbes like viruses and bacterium can even exist as a subclinical reservoir for long periods [1, 3, 4, 9].
7. Hormones such as estrogen increase the severity for breast cancer while estrogen reduced and androgen increased prostate cancer severity [1, 3, 4, 10].
8. Familial DNA genetic alterations where added and passed on from one generation to another [1, 4].
9. Alcohol with more than 2 drinks per day for a long time increases the risk for cancer of the liver, mouth, throat, larynx, esophagus, and breast [1, 3, 4].
10. Diet with high levels of fat can increase cancer risk for the colon, uterus, and prostate. Antioxidant nutrients have alternatively shown decreased risks for cancer [1, 3, 4, 11].
11. Sedentary life style with lack of physical activity is related to overweight factors leading to cancer of the colon, esophagus, breast, kidney, and uterus [1, 3, 4].
12. Hypoxia and ischemia from low molecular oxygen concentrations resulting in mitochondrial free radicals and acid are more general biological conditions that exist for cancer cellular activity [2, 4, 12–16]. Subsequent tumors exhibit anaerobic metabolism that produces energy without oxygen in hypoxic microenvironments [4, 12–16]. Conversely, normal tissues use oxygen for aerobic mitochondrial energy synthesis [1, 12–18].

## 2. Hypoxic Free Radicals and Polymerization

Mitochondria create electrons from different fuel sources of the body that flow through a series of protein enzymes and other mobile electron carriers [17, 18]. These electrons through the mitochondrial electron transport chain combine with molecular oxygen and protons to form water [17, 18]. However, imperfect formation of water by mitochondria during hypoxic low O_2_ states produces free-radical reactive oxygen species (ROS) as superoxide (O_2_^•−^), hydrogen peroxide (H_2_O_2_), and hydroxyl radicals (^•^OH) [15, 18–21]. Other reactive free-radical species can also be formed as well [19]. By definition, a free radical is a molecule that contains an unpaired electron which is highly unstable and seeks out another electron for a stable covalent bond pair [15, 22–24]. Subsequent hypoxic free-radical species are then involved in damage to lipids, proteins, and DNA [15, 18, 19, 24]. As a result, hypoxic low-oxygen concentrations of tumors emphasize the strong influence that free radicals play during the development of cancer such that reactive oxygen species are considered oncogenic [1, 12–16, 20]. 

**Figure 3:**
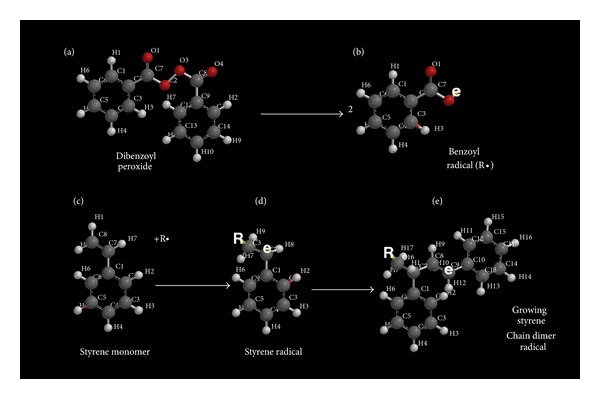
(a) Dibenzoyl peroxide commonly known as benzoyl peroxide or BPO dissociates into two molecules to provide (b) benzoyl free radicals (R^•^). (c) In liquid form, styrene monomer with vinyl-group C7 and C8 (C=C) double pi (*π*) bond upper left of molecule is attacked by R^•^ to add a benzoyl group on one of the carbon atoms. (d) Subsequent styrene radical formation depicted by “e” on the opposite vinyl carbon atom can now enter into a reaction with a new styrene molecule C=C vinyl group. (e) A second styrene molecule can add the benzoyl-styrene radical on the C=C vinyl group for the growing chain to form a free radical again as “e” on the opposite vinyl carbon atom. Free-radical additions through the liquid styrene monomer C=C vinyl groups continue the polymerization process by the reactive secondary sequence method a multitude of times to eventually form a solid.

In terms of similarities with a basic chemistry understanding for molecular pathobiology, polymer science unsaturated alkene chain-growth free-radical covalent bond formation with increased liquid/resin viscosity by molecular crosslinking toward solid structure produces irregular shrinkage patterns [22]. Further, from polymer science free radicals polymerize unsaturated resins to produce increased material modulus (or stiffness) with higher density measured as reduced bulk volume percent from shorter interatomic bond distances [22, 25–27]. In fact, the most characteristic subsequent manifestations of overall polymerization crosslinking is material shrinkage with some internal stresses and warpage [22, 25–27]. A highly common free-radical cure method in past has been a resin system with styrene monomer and dibenzoyl peroxide, depicted in Figure 3, to illustrate free-radical reactive secondary sequence by crosslinking across a carbon-carbon (C=C) vinyl double bond [22]. 

During similar structure-related hypoxic free-radical pathobiology, cell pleomorphism occurs as part of cancer cell EMT by irregular invaginated lipid membranes containing extensive folds associated with possible new lipid polymerization chemistry [22, 28] in the presence of membrane ruffles [4]. EMT pleomorphism further includes extreme differences in cell shapes/sizes [4] related to increased overall tumor mass density from a stiffer (or higher modulus) denser stroma of fibrotissue [4, 29, 30]. In advanced carcinomas EMT-dedifferentiated desmoplastic stroma produces a harder denser overall tumor mass related to a more aggressive grade of cancer that in time becomes an acellular collagenous extracellular matrix [4]. Relative to free-radical covalent bond formation to produce material structure, increasing density surface oxidation crosslinking of rubber reduces oxygen diffusion into the deeper subsurface layers [31]. 

**Figure 4:**
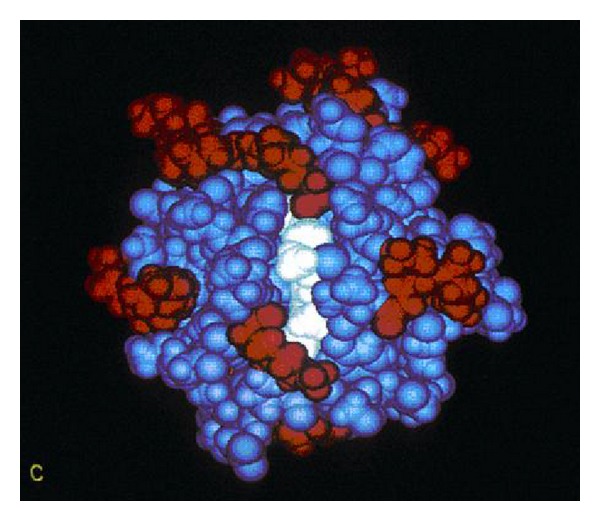
Image depicting cytochrome c which is a small soluble globular protein used in the mitochondrial electron transfer chain that carries electrons as radicals with other mobile transporter carriers ultimately to combine with oxygen and protons to form water [4, 17, 18]. (With permission from the National Institutes of Health/Department of Health and Human Services).

Pertinent to cancer pathophysiology, capillary distance has been measured in tissue showing a respective decrease in both oxygen concentration and pH [4, 14, 32], where increasing hypoxic mitochondrial electron-transport free radicals would be expected to produce some biologic structure from covalent bonding. As increased biologic structure limits O_2_ diffusion more, a new fall in pH with lower O_2_ would be expected by interfering even further with capillary diffusion of molecules. Because oxygen is fundamentally critical to prevent cell death, tumors found over 0.2 mm away from blood vessels fail to grow [4, 14, 32] which is the approximate distance for O_2_ diffusion through living tissue before a zero concentration develops [4, 14, 32]. The low pH eventually becomes an overall result of lactic acid production by anaerobic mitochondria glycolysis energy synthesis [4, 14]. On the other hand, throughout efficient aerobic cellular respiration in the mitochondria, oxygen is fundamental during energy synthesis to form water from the electron respiratory transport chain and proton gradient [21, 33–35]. Part of mitochondrial respiratory aerobic energy synthesis includes one of the most well-studied enzymes ever with cytochrome c that was identified in earliest National Cancer Institute research, Figure 4. The enzyme cytochrome c is part of the electron transport chain and localizes in the intermembrane space but can leak into the cytosol to participate in cell death by apoptosis with the caspase enzymes [1–4].

During hypoxia, cells switch to anaerobic glycolysis to produce lactic acid [14], but with alternate aerobic respiration energy synthesis reactive oxygen species with other free radicals and also protons start to form [21, 33–35]. However, cancer cells can adapt to hypoxia by high glucose uptake with anaerobic glycolysis and lactic acid production to minimize free radical formation from the mitochondria [4, 14, 16, 36]. Subsequent hypoxic cancer conditions then not only produce large amounts of free radicals [4, 15] but also contribute to acid formation for a much lower pH microenvironment [4, 14, 16, 36]. Through a mechanism known as the Warburg effect [14, 16, 36], cancer cells continue to produce energy by aerobic glycolysis even with O_2_ present [14, 16, 36] possibly as mentioned before as an adaptive mechanism to help limit hypoxic free-radical formation by the mitochondria [36]. Free radicals may represent a potentially more serious threat than lower pH since free-radical molecular structure through the rapid chemistry of covalent crosslinking limits oxygen diffusion progressively as a primary source for cancer pathology through continual increasing levels of free radicals and acids from oxidatively stressed mitochondria.

In addition to chronic infections with widespread inflammatory cells, chronic inflammation has been recognized as a major common cause of cancer promotion that further involves free radicals [3, 4, 19, 37, 38]. Sources of chronic inflammation can include microbial/viral infections or toxic/allergic substances and also obesity [3, 4, 19, 37, 38]. Dysplasia occurs in chronic inflammation and also in benign growths before premalignant cell proliferation [4, 37, 38]. Cells that invade through the basement membrane are then considered malignant [4, 37, 38]. Pleomorphism occurs as a sign of dysplasia with cells displaying large variations in cell sizes and shape morphologies with unusually large deeply stained nuclei [4, 37, 38]. Mitotic cellular division is also high such that the increased possibility of malignant transformation through EMT can occur by benign cells unrestrained from normal process in the dysplastic states [4, 37, 38]. In addition, inflammatory cells mediate cancer progression through free radicals that can mutate DNA, produce epigenetic alternations with DNA methylation, increase cell proliferation, oxidize lipids, crosslink proteins, and promote angiogenesis [4, 19].

## 3. Other Important Fundamentals in the Study of Cancer

**Figure 5:**
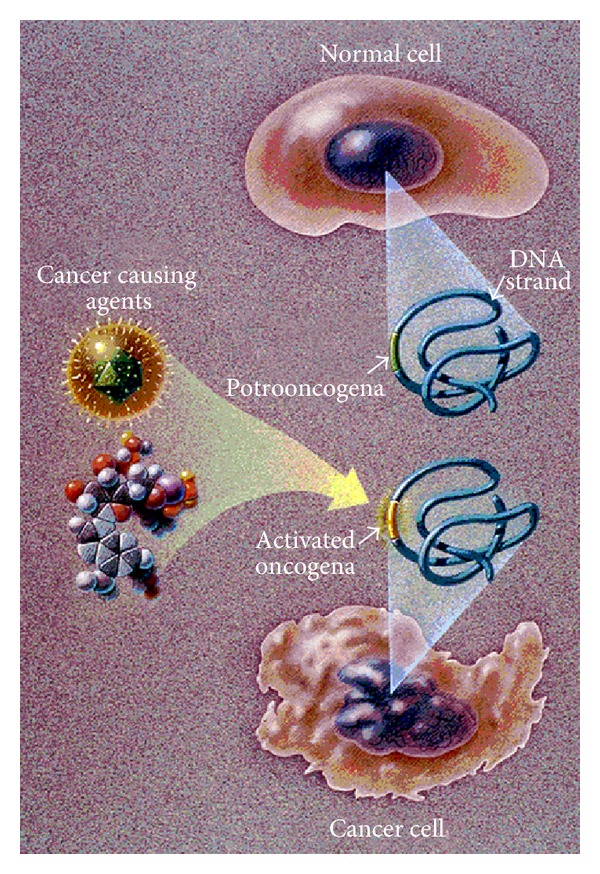
National Cancer Institute illustration shows the stages of how a normal round cell is converted to a cancer cell with shape irregularities for both the nuclear and plasma cell membranes when an oncogene becomes activated. (With permission from the National Institutes of Health/Department of Health and Human Services).

1. Angiogenesis or blood vessel development was shown to be an important part of tumor expansion that could be used as a pharmaceutical chemotherapeutic anticancer target. Later research showed the pathology that tumors produce factors to encourage angiogenesis with new abnormal blood vessels to support cancer growth. Tumors cannot increase to more than 1-2 mm in diameter without growing blood vessels to bring in oxygen and nutrients [2, 4].
2. Apoptosis is a normal cell physiologic death that helps to prevent unwanted cell growth. However, tumors on the other hand contain cancer cells with mutations that prevent normal apoptosis. Cells lacking apoptosis death regulatory pathways are then more resistant to death in addition to chemotherapeutic cancer cytotoxic agents [4, 39].
3. Tumors secrete protease enzymes that degrade collagen protein to provide exits for entering the blood stream and metastasizing to more distant tissue sites [2, 4, 14, 40].
4. DNA methylation can turn a gene off [3, 4, 41–44]. Hydrogen abstraction has been commonly identified as part of a free-radical lipid peroxidation breakdown process that produces characteristic biomarkers. Since breaking a carbon-carbon bond requires much less energy than breaking a hydrogen-carbon bond [22, 23], highly reactive methyl and other small acyl-free radicals could be considered as sources for DNA methylation during periods of lipid peroxidation or during hypoxia with the generation of free-radical electrons and acid [22].
5. Tumor suppressor genes inhibit cell proliferation and are targets of carcinogenesis when such genes are inactivated. For example, proteins from several viruses bind to a tumor suppressor gene to stop manifestation of downstream functional activities to subsequently promote cancer. The most common tumor suppressor gene is the p53 gene that initiates apoptosis through the p53 protein [4, 45–47].
6. Protooncogenes code for cell growth or proliferation and can mutate to become oncogenes to cause cancer, Figure 5. Common proto-oncogenes can code for growth factor proteins, cytoplasmic enzymes, membrane receptors with tyrosine kinase enzymes, or transcription factors in the nucleus for cell division [4, 48–50].

## 4. Irregular Membrane Ruffling by Free-Radical Crosslinking

In order to better simplify cancer fundamentals into a molecular biology polymer approach for an easier mechanistic understanding theory of cellular and tissue changes during carcinogenesis, figures in imaging from the National Cancer Institute are helpful. A typical National Cancer Institute consensus illustration, Figure 5, depicts the basic morphology that a cancer cell has irregular borders with membrane wrinkling as ruffling when compared to the identical normal rounded cell. In addition, Figure 5 shows how an oncogene is activated by a cancer causing agent so that instead of normal cell division, oncogenic cellular DNA damage causes cancer. Cancer cell nuclei are also misshapen into strange unusual irregular shapes compared to the normal rounded oval nuclei with smooth borders. For a plausible explanation regarding the membrane irregular borders with ruffling in cancer cells, free radicals need some understanding. As a constant source, free radicals produced during hypoxia are found at high levels in cancer cells [1, 12–16]. Further, excessive free radicals as reactive oxygen species attack unsaturated fatty acids found as lipids in cell membranes with carbon-carbon (C=C) double bonds [22, 24]. 

One of the most characteristic features for free-radical covalent bonding in a liquid to increase viscoelastic solid structure with increased modulus and density properties becomes most apparent in materials that polymerize by electron-pair chain-growth polymerization as the linear/volumetric cure shrinkage [22, 25–27, 51, 52]. In fact, free radicals are engineered for specific materials science application to crosslink molecules with consequent cure shrinkage and possibly warpage as one the most distinguishing material problems of extensive polymer electron-pair bonding [22, 51, 52]. Because shrinkage is not necessarily perfect with inhomogeneous material, nonuniform electron pairing in curing in addition to increasing the modulus can create residual internal stresses to produce warpage of materials that weakens parts [51, 52]. Warpage is particularly accentuated during free-radical polymer curing with thin film coatings of variable thickness without good support [52]. As covalent single sigma (*σ*) bonds form by replacing C=C pi (*π*) bonds during reactive secondary sequence chain growth [22, 25–27], polymer chains draw together from more distant van der Waals intermolecular attraction forces and chain-entanglement equilibrium distances to closer covalent distances with increasing chain entanglement that reduce bulk volume by linear/volumetric cure shrinkage [22, 25–27]. Free-radical double-bond conversion to single bonds is thermodynamically favorable and forms an exothermic polymerization even without extra energy added at room temperature [51]. Consequently, solutions of unsaturated lipids that undergo thermoset free-radical chain growth will also produce linear/volumetric cure shrinkage without added energy [22, 25–27, 51, 52].

**Figure 6:**
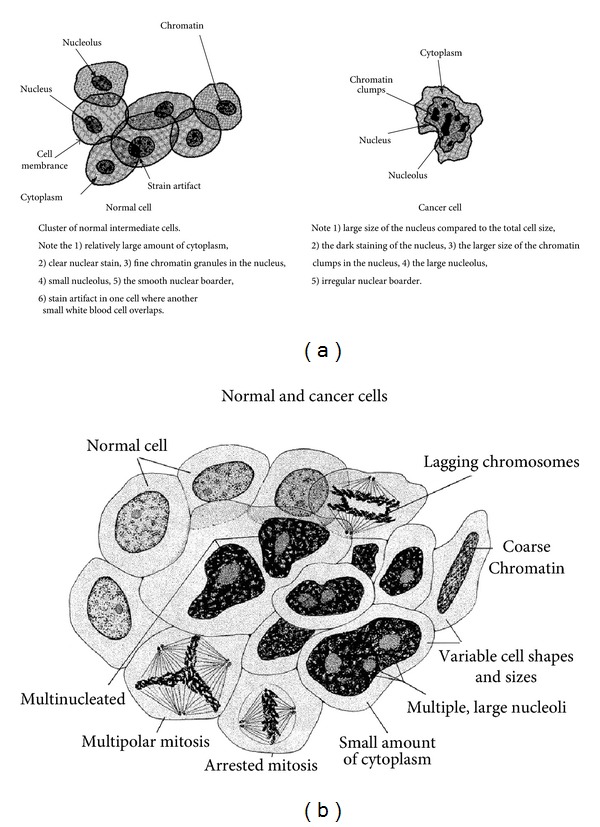
(a) Graphics and description comparing normal round cells and cancer cells with both irregular nuclear and plasma cell membranes (With permission from the National Institutes of Health/Department of Health and Human Services). (b) The round normal and cancerous characteristics with warped irregular borders are identified. (With permission from the National Institutes of Health/Department of Health and Human Services).

**Figure 7:**
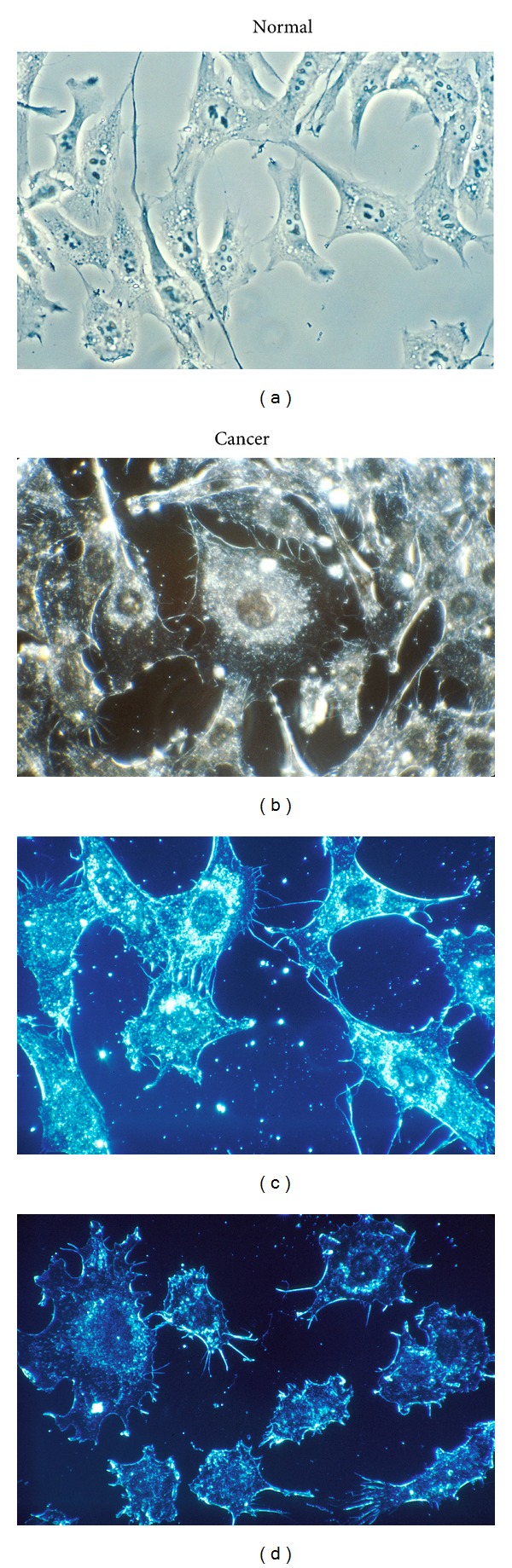
Normal cells on the left and cancer cells with more spike-like membrane extensions on the right in culture from human connective tissue. At a magnification of 500x, the cells were illuminated by darkfield amplified contrast technique. (a) Normal cells compared to (b) cancer cells. (b) Normal Cells compared to (d) cancer cells (with permission from the National Institutes of Health/Department of Health and Human Services).

Cytoskeleton actin fibers provide tensile strength and support to the cell [4] so that the composite plasma cell membrane with lipid oils and phosphate groups offers conditions that maintain separate mediums to accentuate free-radical polymerization warping particularly as an outer veil thin film. Hydrocarbon lipid molecules drawn together at a rounder border would require some invagination to wrinkle inward especially when combined with coupling to underlying rigid fibers resulting in the possible explanation for common ruffling irregular membrane appearances of cancer cells depicted in consensus National Cancer Institute Figures 5, 6(a), 6(b), and 7(a)–7(d). Lipid oil with an unsaturated linoleic/oleic fatty acid combination for free-radical crosslinking has previously demonstrated a wrinkling and warpage during a solidification polymerization process [22]. As well as irregular plasma cell membrane borders on the outer periphery of a cancer cell, the nuclear membranes are misshapen and nuclear to cytoplasm ratios increase [36, 37]. In addition to normal cell round morphology changing by EMT to irregular membrane border patterns with invaginations in cancer, other common findings are presented as basic consensus for changes in cancer cells through the National Cancer Institute with two separate illustrations, Figures 6(a) and 6(b), as.

1. darker staining nucleus,
2. coarse chromatin and clumping,
3. more irregular nuclear border,
4. less cytoplasm and larger nuclei,
5. multinucleated,
6. variable pleomorphism in cell sizes and shapes.

Cell cultures that show normal cells with smoother membrane outlines compared to cancer cells with more irregular membranes help to document the plasma cell membrane spike-type extensions that form deeper invaginated irregular borders as part of the EMT with transformation to cancer, Figures 7(a)–7(d). 

**Figure 8:**
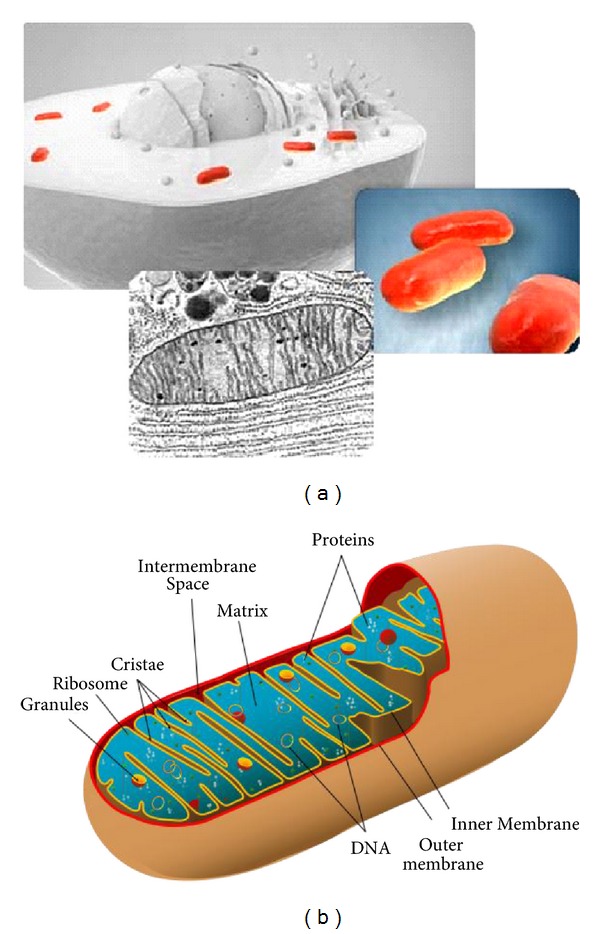
Mitochondrial inner membrane cristae (a) 3D illustration upper left shows spheroidal mitochondria in red located peripherally circumferential around the nucleus and SEM below showing convoluted cristae. (b) Mitochondrion illustration with tortuous cristae ((a) with permission from the National Institutes of Health/Department of Health and Human Services).

Another more common intracellular organelle membrane feature possibly related to covalent C=C double-bond crosslink shrinkage and thin-film warpage [52] includes the most distinctive inner membrane features of the mitochondria with the high convolutions called cristae [17, 18, 33–35], Figures 8(a) and 8(b). The convoluted mitochondrial cristae that make up the inner membrane serve to create an impermeable confining larger surface area for important biologic electron interactions during aerobic respiratory energy synthesis [17, 18, 33–35]. Subsequent electron flow during energy synthesis [17, 18, 33–35] should further be an extreme source for free-radical lipid crosslinking through C=C double bonds to provide the structural convolutions noted in the mitochondria. The mitochondrial interior inside the inner membrane is a gel of approximately 50% protein [18]. Most of the mitochondrial protein studies thus far are not fibers but rather soluble or globular enzymes [17, 18, 33–35] that would not provide cytoskeleton-type support like structural fibers in a composite [51]. During crosslink shrinkage by high concentrations of free radicals from the electron transport chain, the lipid hydrocarbons would accentuate warpage into convolutions of the thin-film inner membrane without the more stable influence from reinforcing stacked planar fibers provided by the plasma cell membrane or nuclear membrane. 

Proteins contain ionizable groups on the terminal carboxylic acid end, the terminal amino end, and many side chain groups to function as buffers [18]. Proteins also generally exist with a negative charge at physiologic pH 7.2 [18]. Since proteins maintain a negative charge intracellularly and are further the major blood buffer as part of the plasma [17], the ideal medium is available to sequester radicals from the mitochondrial electron transport chain and buffer protons from the proton gradient in the form of the multiple protein enzymes that help synthesize energy aerobically by oxygen with water as the final product. In fact, amino acid side chains on hemoglobin protein [53] and even in short peptides [54, 55] are known to sequester radicals with tyrosine, histidine, and cysteine residues [54, 55]. Further, radicals can be delocalized from a side chain into the peptide bond [54, 55]. Although the spin density for radical delocalization from a side chain into the peptide bond atoms is small for pentapeptides, projections could be large depending on peptide bond conformational changes or electrostatics [55]. In addition, proteins could increase radical delocalization through the much higher numbers of peptide bonds than a small peptide molecule so that radicals from the electron mitochondrial transport chain could be stabilized for even longer periods than normal radical intermediates studied thus far. So, instead of radicals disseminating into different pathologies constantly, much longer induction periods would be made available by proteins acting as antioxidants, but nevertheless could still become oversaturated eventually to act as an electron pool or source for free-radical damaging properties. Still, free-radical crosslinking of the mitochondrial inner membrane would help explain the extreme convolutions that structure into an impermeable medium.

## 5. Cell Movement and Lamellipodia 

**Figure 9:**
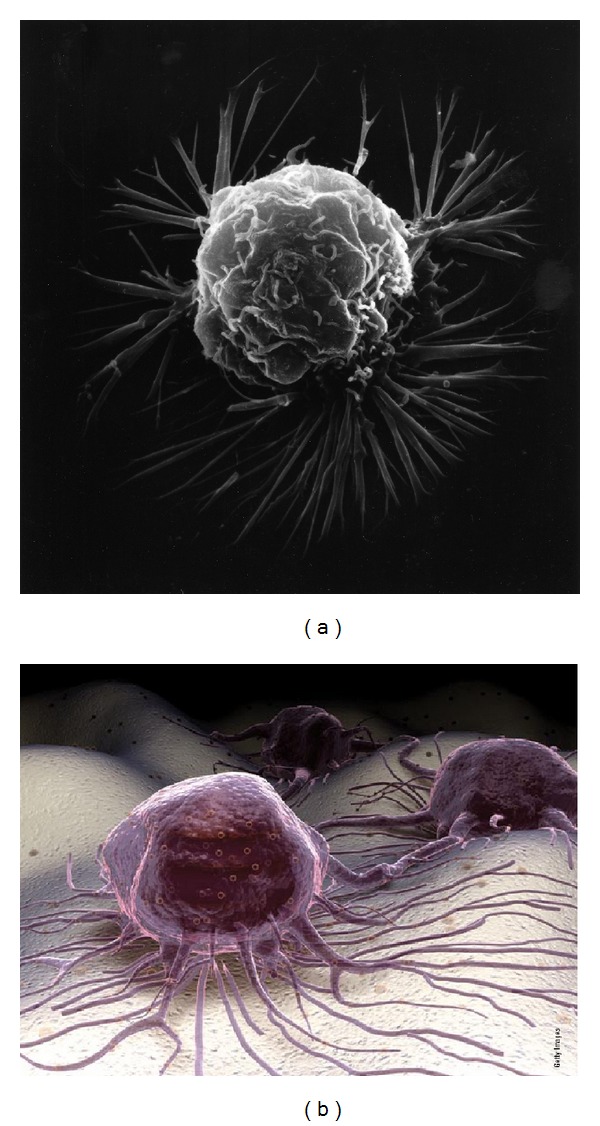
(a) SEM of a breast cancer cell that clearly shows both the soft petals of membrane ruffling and much longer lamellipodia spiking extensions. (b) A scanning electron microscopic 3D-enhanced NIH image of cancer cells and lamellipodia spike processes on a cellular tissue surface (with permission from the National Institutes of Health/Department of Health and Human Services).

A state-of-the-art scanning electron microscope (SEM) shows the intricate details for irregular plasma cell membrane borders that form ruffling and an extensive network of structural spike-like ridges that project long distances, Figure 9(a). A more complete conception of an SEM 3D-enhanced National Cancer Institute image shows how a cancer cell and its long processes called lamellipodia move on a cellular tissue surface, Figure 9(b). Carcinogenesis requires cell movement with EMT and cell shape changes for metastasis and invasion [2, 4, 56]. Subsequent cell movement involves reactive oxygen species that includes H_2_O_2_ as common denominators for the formation of the hydroxyl-free (^•^OH) radical which in turn creates protrusions at the cell edges [57, 58]. Cell movement is directed through extracellular chemical gradients by chemotaxis [2, 4, 59]. Free radicals that form as a constant source from reactive oxygen species including H_2_O_2_ have been shown to act as key chemotactic factors to regulate chemoattractants that bind to cell membranes with actin polymerization for cell migration toward H_2_O_2_ and other reactive oxygen species [60–62]. Further, the traveling cell is polarized by microtubules radiating from the centrosome near the nucleus to the outer cell edges [63, 64] to grow actin protrusions with adhesions between the extracellular matrix that contract in the forward direction [4, 57, 63, 64]. The cell protrusions are long lamellipodia extensions and short focal adhesive filopodia constructed from actin fibers that polymerize at the advancing forward edge [4, 57, 64]. Conversely, depolymerization of actin occurs away from the advancing cell edge [4, 57, 64]. Cytoskeleton microtubule and actin fibers are polarized positively near the cell membrane to elongate [57, 64] and negatively by microtubules toward the organizing centrosome center near the nucleus [56, 65]. A strong long-range static electric field develops on the mitochondria and also on the microtubules that lie in close contact [66] resulting in a possible delocalization mechanism for the electron transport chain during periods of mitochondrial oxidative stress. Polymerization of actin fibers at the plus end forms protrusions that contain focal adhesions with the extracellular matrix so contractions provide forward movement [4, 57, 63, 64]. In addition, depolymerization occurs at the negative ends of the actin fibers on the rear edge of cell movement for release of focal adhesions and in addition making actin monomers available to be recycled for polymerization at the forward positive actin protrusive end [4, 57, 64]. 

Free-radical crosslinking and agglomeration with weaker secondary bonding previously described account for atoms, molecules, and larger molecular chains being drawn together [22, 25–27, 51, 52] to provide a possible mechanism for forward movement in the contraction process. Lipid peroxidation products with low molecular weights and a C=C double bond have demonstrated high crosslinking with both unsaturated fatty acids [22] and protein [67]. Further, a polyunsaturated fatty acid with 6 C=C double bonds has been shown to activate cells as a chemoattractant to migrate during reorganization of actin fiber in lamellipodia using a strong free-radical oxidant as peroxinitrate [68], with strong free-radical crosslinking indication for contraction through covalent bond shrinkage [22]. In addition to lipid free-radical crosslinking across C=C double bonds as a reactive secondary sequence, amino acid side chains of protein are modified at about 50 percent of the residue by the hydroxyl-free radical formed from H_2_O_2_ [69]. Further, proteins are found to agglomerate or crosslink most notably through the amino acid tyrosine [70, 71] with metal-catalyzed reactions [70].

While most cancer cells studied through the National Institute of Health figures presented demonstrate extensive spike protrusions that could greatly interfere with movement and prevent leakage through a small tissue pore, the more aggressive cancer cells have a smaller membrane area that allows the lower modulus cells to more easily squeeze through and penetrate small openings in the endothelium [72]. Nevertheless, membrane protrusions are most frequent on the leading edge of cancer cells during metastasis movement [73]. Formation of the leading edge with cytoskeleton actin fibers that shape lamellipodia [4, 73] is only about 200 nanometers thick [4, 74, 75]. In addition, the actin fibers orient along the axis of the membrane protrusions so that the modulus is highest to resist deformations in the lamellipodia plane and much lower in the perpendicular plane [73]. As a result, the leading lamellipodia edge can push with leverage through the smallest openings and still deform on the sides to start squeezing through extremely narrow spaces for escape into new tissue [73]. Fiber polarizations from the negative centrosome end near the nucleus to the positively charged plasma cell membrane side [63–65] are thought to be responsible for the forward polymerization of the actin fibers during cell movement [57, 63, 64]. As such, electrons conducted from microtubules into actin fibers by highly charged mitochondria [66] overstressed under hypoxic conditions should accumulate at accentuated levels through the electron transport chain deprived of oxygen that could help account for a portion of the free radical polymerization mechanisms with actin fibers. In fact, actin has been shown to restructure under free radical conditions with H_2_O_2_ to increase cell motility [76]. Regarding H_2_O_2_ ability to polymerize actin by free-radical chain lengthening mechanisms, H_2_O_2_ has proven to be an exceptional initiator for free-radical polymerization in polyester resin formulations [77]. By related free-radical reactive oxygen species biological chemistry, actin polymerization has also been observed in macrophages exposed to oxidized low-density lipids [78]. Further, high levels of H_2_O_2_ and other reactive oxygen species are found in various cancer cells [79].

## 6. Nucleus Changes with Free-Radical DNA Methylation

Cancer initiation and progression have been linked to increasing free-radicals following oxidative stress and reactive oxygen species that are continuously produced by mitochondria during cell metabolism [4, 15, 79–81]. Cancer cells are characterized by increased reactive oxygen species and accumulation while the mitochondria are considered the major source for reactive oxygen species [81]. Subsequent hypoxic mitochondrial free-radical species are then involved in damage to lipids, proteins, and DNA [4, 15, 18, 19, 24, 81]. As a result, hypoxic low oxygen concentrations of tumors emphasize the strong influence that free radicals play during the development of cancer such that reactive oxygen species are considered oncogenic [1, 12–16, 20, 81]. A major role for free radicals produced by hypoxia or ischemia through mitochondrial metabolism includes crosslinking with either simple electron pairing or as extensive reactive secondary sequence C=C double-bond chain growth. A major effect of free-radical crosslinking molecules is a characteristic volumetric shrinkage and warpage [22, 25–27, 51, 52]. By similar free-radical crosslink chemistry, biologic crosslinking could explain the coarse or clumping chromatin depicted in Figures 6(a)-6(b), coupling of DNA to DNA or DNA to protein [15], abnormal cell shrinkage with warpage and irregular membrane borders [4, 37, 38, 59, 66], membrane ruffling [4], protein agglomeration with insoluble accumulation [15, 70, 71], and actin polymerization [76]. Also, as the nuclear membrane structures, a greater amount of the cell is constrained within the nucleus [37, 38, 59]. 

In addition to genetic DNA alterations or changes in the DNA sequence [4, 14], epigenetics that alter the gene slightly further influence cancer growth and metastasis [4, 80]. Hypoxia is already accepted as a general condition that promotes tumors [14, 82, 83], supports cancer recurrence [83], intensifies malignancy [14, 82, 83], increases metastases [14, 83, 84], and inhibits chemo/radio therapies [14, 83]. The main epigenetic change is by DNA methylation that can be linked to hypoxic-free radical environments with acyl-radical breakdown products [4, 22, 41–44, 85]. Molecular breakdown is further a well-known biomarker when free radicals accumulate with unsaturated lipid fatty acids [86–89] especially when combined with lower pH acid [22] that occurs during hypoxia in mitochondrial metabolism [22]. Further, thermodynamics for bond dissociations studied for lipid peroxidation favors acyl radical formation over hydrogen bond dissociation as a key source for DNA methylation [22]. Hypermethylation of DNA subsequently causes gene silencing of particular importance for tumor suppressor genes [4, 80]. Oxidation of DNA is another risk factor for mutation that modifies bases to favor cancer [4]. Subsequent long-term stages of hypoxic-related free radicals particularly seen with chronic inflammation then account for advanced chromosomal damage with DNA base substitutions particularly through purine replacement by a pyrimidine termed a G→T transversion, DNA crosslinks, and chromosomal breaks [4].

## 7. Metastasis

**Figure 10:**
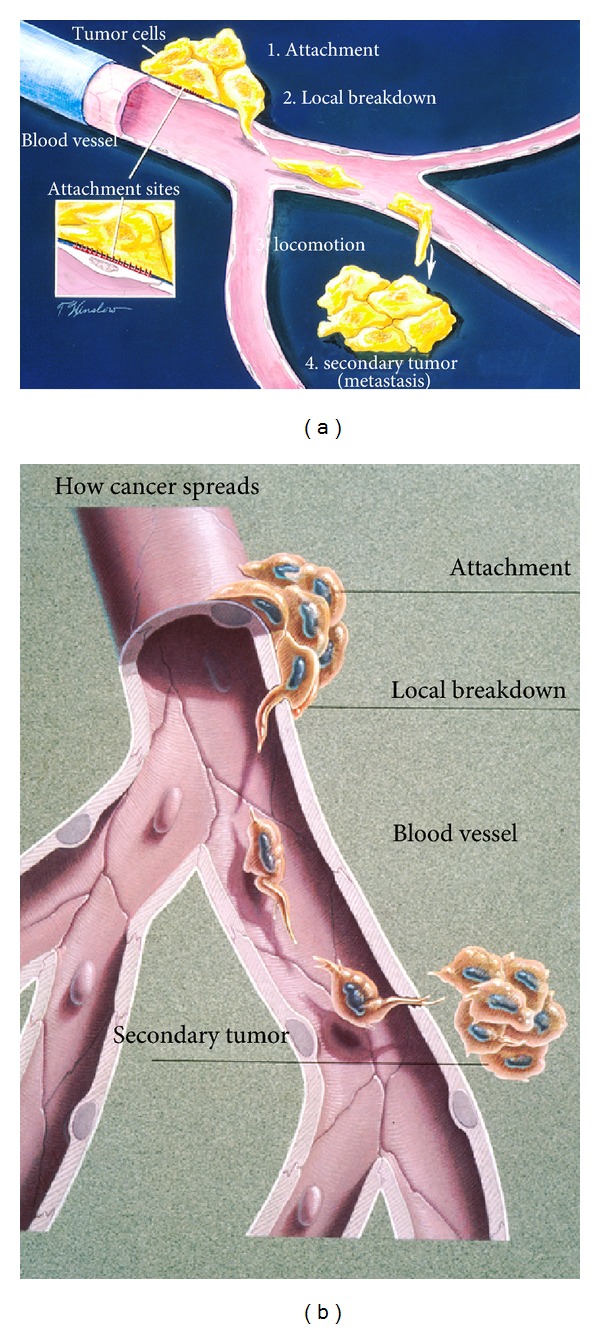
(a) Schematic drawing for the stages of metastasis (1) attachment (2) local breakdown with lamellipodia penetration into a blood vessel (3) locomotion with lamellipodia to exit from blood vessel (4) secondary tumor. (b) Once metastatic cells are attached to the vessel wall basement membrane (a physical barrier that separates tissue components), cancer can break through with stiff lamellipodia on the leading edge and the help of protease enzymes. Cancer cells then move through the blood stream enabling them to spread to other parts of the body. A secondary tumor may subsequently form at another site in the body. (With permission from the National Institutes of Health/Department of Health and Human Services).

Although a cancer tumor can invade adjacent tissue directly, most cancer deaths occur by metastasis to distant tissue through the blood [2, 4, 14]. Metastasis takes place through an extremely biocomplex sequence of events in several stages, Figures 10(a) and 10(b). Biocomplexity is a hallmark of cancer that involves both a low pH acidic environment [4, 14] and free radicals [4, 15, 79–81] produced from hypoxic conditions [4, 14, 82–84, 90]. Metastasis further involves cell motility with EMT cell shape changes [4] and also destructive tissue breakdown by pH-activated protein enzymes [4, 14, 91]. Further, focal contacts develop between the extracellular matrix and filopodia that extend as short spikes off the advancing lamellipodia lengthening arms [4]. Microtubules polarized by electric fields influenced through close association with mitochondria [66] have capability to conduct electrons or transport free radical species rapidly to the plasma cell membrane. Free radicals available as a result of low oxygen concentrations leaking from the electron transport chain can then help to provide adhesive bonds by electron pairing and secondary bonding forces during cell migration through focal contacts with the filopodia. In fact, unsaturated lipids that have C=C double bonds with reactive secondary sequence capability [22] are known to influence cell adhesion by focal contacts with the extracellular matrix [92]. 

The first advanced invasive stage involves attachment of cancer cells from the primary tumor to the basement membrane of local blood vessel endothelial cells [4]. Endothelial cells compose a single layer that wraps around to form a tube inside the blood vessels which are further depicted in Figure 10(b). The next stage involves local breakdown of the endothelial cell barrier by cancer cell protease enzymes filled with both free radicals and acids [4, 93, 94]. As a related biocomplex event, the oxidation of the amino acid cysteine by reactive oxygen species H_2_O_2_ includes disulfide crosslinks that are involved with metastasis and reduced enzyme activity [95]. 

**Figure 11:**
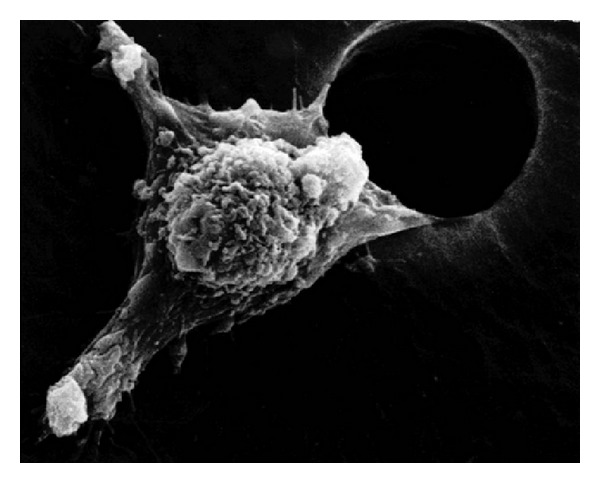
SEM of a cancer cell movement through a man-made hole involves “arms” or pseudopodia, termed lamellipodia, enabling them to migrate to other parts of the body. Locomotion cell motility is integral to the entire process of invasive metastasis. (With permission from the National Institutes of Health/Department of Health and Human Services).

Cancer cells with greater metastasis potential have a lower modulus and lower viscosity than normal cells with the ability to deform more in addition to the pleomorphic smaller sizes with less cell membrane area [50, 91, 96]. Cell stiffness increases with organized actin fibers of the cytoskeleton and during cancer transformation actin fibers are disrupted into irregular networks that results in a lower modulus or less stiff, more deformable cell [96]. Conversely, overall increased tumor tissue density is a risk factor for cancer [30, 97, 98]. Increasing stroma density is further characterized by increased collagen deposition [98] that provides better traction forces with focal adhesions to promote cell migration for metastasis [30, 99]. Also, cells tend to migrate toward stiffer substrates [99]. Through a similar reference to cell structure, cancer cell pseudopods with high modulus actin fibers [4] provide stiff leverage movement through narrow gaps to invade other tissue, Figure 11. But, in opposition again with another molecular biology reverse structural-related mechanism, tissue degradation from cancer hypoxic-associated lower pH with protease enzymes removes intercellular adhesion to more easily release cells from the primary tumor and to carve out space for invading cancer cells [4]. Consequently, the smaller cancer cells with lower moduli can subsequently be freed from the primary tumor to move through small openings [100] produced by the common protease breakdown and enter the blood stream. On the other hand, as larger cancer cells invade, metastasis then most commonly progresses when cells get trapped in capillaries to spread into new tissue [4].

## 8. Protease Enzymes

Cancers revert by EMT to more primitive less differentiated cells with increased acid production [4, 14]. Subsequent protease enzymes activated by acid due to related hypoxic conditions are responsible for degrading structural fibers of the extracellular matrix [4, 14, 91]. As the extracellular matrix is broken down under hypoxic conditions at a much lower pH than normal, cancer cells can initiate paths through the extracellular matrix for cancer cell migration to spread and eventually detach into areas occupied by dying cells [4, 14]. Cancer cells can eventually even use the surrounding dead cells for a nutrition source during rapid cell proliferation by intracellular means with oncogene expression of enzymes linked to membrane receptors [4]. Further, in an unusual biocomplexity, cancer cells are able to induce stromal cells such as fibroblasts to produce H_2_O_2_ reactive oxygen species and lactate acid through metabolic anaerobic glycolysis to increase invasiveness [4, 14, 72]. In addition, although the pH of the interstitial fluid in the extracellular matrix surrounding a cancer tumor is lower than physiologic pH, the cancer cytosol is either neutral to slightly basic so that cancer cells develop a stronger advantage over the surrounding normal cells in the stroma [14]. Since chronic inflammation is a major cause of cancer [19, 85], inflammatory cells attracted to dead cells surrounding the cancer network would then be expected to increase free radicals further above the high levels observed in hypoxic cancer tissues [79]. 

Cancer cell adaptation to high free-radical concentrations and the extreme lower pH in the surrounding extracellular matrix at a much superior level to normal cells [4, 14] must require buffering. Since proteins delocalize radicals [101] and generally exist with a negative charge at physiologic pH 7.2 [18] to maintain a negative charge of protein intracellularly [17] and further are the main buffers in the blood [17], increased oncogene protein expression at the plasma cell membrane level [4] might explain superior cancer cell buffering. Of particular interest in buffering low pH extracellular matrix microenvironments are protein kinase enzymes in the cytosol associated with hydrophobic protein membrane receptors [4]. In fact, the majority of cancers are associated even through intracellular protein kinase to membrane receptor ligand binding with the extracellular matrix by phosphorylations on intracellular tyrosine amino acid residues [4, 102, 103]. Phosphate then is the important recognized intracellular buffer [17]. Thus, the cancer cell can be protected from harsh low pH microenvironment conditions with excellent membrane receptor buffering systems that respond rapidly through protein radical delocalization in addition to counteracting high acid concentrations from the extracellular side of the plasma cell membrane. 

Membrane receptor signaling into the cell from the extracellular matrix is subsequently associated with EMT cancer progression that changes cell shape, promotes proliferation, and increases cell motility for invasive properties [4, 102, 103]. Subsequent rapid molecular signals could be transferred by proteins at the outer cell membrane into the cell in the form of instantaneous radicals and low molecular weight acids by relative fast mechanisms. Tyrosine kinase membrane receptors then not only act as ligands for binding growth factors and extracellular matrix [4, 102, 103], but further provide intracellular kinases [4, 102, 103] for enzyme activity. Tyrosine kinase enzymes linked to membrane receptors would be able to efficiently continue the breakdown of molecules already being degraded across the membrane in the extracellular matrix at low pH as nutrition sources for cancer with increasing well-organized catalytic nutritional mechanisms. In addition, oxidative mechanisms by reactive oxygen species exist at low pH in the extracellular matrix to dissociate C=C bonds on lipids that might normally be difficult to disrupt [22].

During the extravasation process where cancer cells escape the blood vessels [4], protease enzymes released into the blood stream or incorporated in metastatic cancer cells as a continuation of the original EMT process [4, 102, 103] might be expected to assist the breakdown of endothelial cells at distant locations. The low modulus cancer cell can then deform to squeeze and push through small openings with high-modulus protrusions into distant tissue sites. Although invasive cancer cell movement requires proteases for degradation of the extracellular matrices forming tube-like tracks for lamellipodia extensions, subsequent actin-protein-like buffering by the lamellipodia with cell rounding allows more extensive amoeba-type movement in matrix gaps [104]. When cancer cells enter the lymphatic system, the metastasis occurs in the lymph nodes or continues on to the blood stream [4]. Cancer cells can further become trapped in capillaries and enter organs to form the secondary tumor [4]. Because cancer cells are pleomorphic and contain much larger and much smaller cells than normal, some larger cancer cells become trapped in capillaries [4], while smaller cells can easily enter even more distant sites through openings created possibly by tumor proteases. 

## 9. Cancer Chemotherapeutic Research 

**Figure 12:**
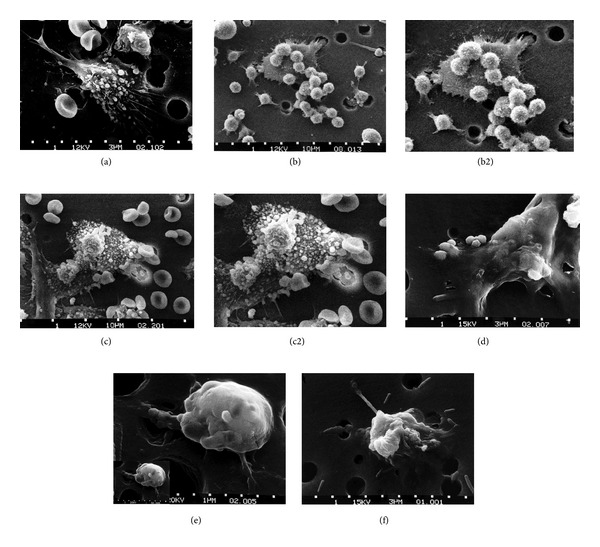
Six-step sequence showing the death of a cancer cell. (a) One cancer cell is migrating through a hole of a matrix-coated membrane from the top to the bottom, simulating natural migration of an invading cancer cell between, and sometimes through, the vascular endothelium. Notice the spikes or pseudopodia that are characteristic of an invading cancer cell. A film coat containing red blood cells, lymphocytes, and macrophages is added to the bottom of the membrane. (b) A group of macrophages identify the cancer cell as foreign matter and start to stick to the cancer cell, which still has its spikes. (b2) enlarged for relative cancer cell size comparisons for approximate equal magnifications between Figures (a), (b2), (c2), (d), (e) inset lower left, and (f). (c) Macrophages begin to fuse with, and inject toxins into, the cancer cell. The cancer cell starts rounding up and loses its spikes. (c2) enlarged for cancer cell size comparisons. (d) As the macrophage cells become smooth, the cancer cell appears lumpy in the last stage before it dies. (e) Lumps covering the cancer cell surface are actually the macrophages fused within the cancer cell with inset at lower left for cancer cell size comparisons showing a great reduction in size. (f) The cancer cell then loses its morphology, shrinks up more, and dies. (With permission from the National Institutes of Health/Department of Health and Human Services).

A high percent of cancers metastasize or occur within inoperable tissue that result in the majority of deaths [4, 14] and are untreatable by surgical incision so that radiation treatment or chemotherapy is necessary [4, 14]. Chemotherapy is often considered because cancers proliferate faster than normal cells so that drugs interfering with fast growing tissue cells should eradicate cancer cells [14]. However, treatment may aggravate the tumor microenvironment and make cancer cells worse [14]. Further, because chemotherapeutic agents must kill cancer cells that survive beyond apoptotic events, toxicity is extremely high to normal cells [14]. To place cancer chemotherapeutic treatment in better perspective concerning killing cancer cells, images showing cancer cell death can help, Figures 12(a)–12(f). 

An immune response by many sacrificial macrophages is required to kill a single cancer cell *in vitro*. Once the cancer cell is detected, macrophages will attach to the larger cancer cell to absorb the hypoxic mitochondrial metabolic wastes of acid and free radicals and reinject such toxins back into the cancer cell. The macrophages then die with the cancer cell. However, as malignant tumor proliferation becomes successful, cancer cells can use macrophages, or tumor associated macrophages (TAMs), to help destroy normal cells with proteases and further assist tumors by bringing in endothelial cells, capillaries, and needed oxygen as part of the cancer growth and invasive process [4].

## 10. Regenerative Medicine Pharmaceutical Prodrugs 

From the free-radical polymer-based cancer model developed, a new class of safe and effective regenerative medicine prodrugs could evolve that reduce the original problems associated with hypoxic conditions [4, 14, 85–88, 90] by mitochondrial free radicals [4, 12, 15, 79–81] and acid [4, 12, 14, 72]. Further, cancers are noted to have a measurable acidic extracellular matrix microenvironment [4, 14, 72]. Although reactive oxygen species are thought to promote cancer [4, 15, 35, 79–81], clinical trials using vitamins to reduce the free radicals associated with cancer suggest that nutrient antioxidant supplements are not effective in reducing risks during treatment [105, 106]. Problems already identified with nutrient supplements include high reactive secondary C=C crosslinks with vitamin A and *β*-carotene and nonantioxidant activity by vitamin E that may possibly provide alternative benefits as a viscosity reducer of lipids [22]. Due to poor efforts in reducing the pathology of cancer by vitamin therapy, antioxidants are not recommended during cancer treatment so as not to offset chemotherapeutic free-radical-related killing toxicity [105].

Free-radical inhibitors engineered to prevent premature crosslinks in polymer resins and highly active monomers [22, 107] could be initiated as adjunctive treatments with medical treatments for cancer. In fact, the hydroquinone/benzoquinone system has already been used in the prodrug treatment of hypoxic tumors [108]. Free radicals produced under tumor hypoxic conditions that create structures at the molecular, fiber, cell, and pathological tissue levels would not only increase the vicious cycle of hypoxic pathology by interfering with oxygen deliver but also interfere with pharmaceutical drug transport during treatment. In addition, free radicals are involved in the polymerization process for actin fiber development that creates protrusive lamellipodia structures for advanced EMT cancer cell movement, tissue invasion, and vascular metastasis [57]. Further, the hypoxic tumor environment produces high levels of free radicals that have been implicated in angiogenesis [109].

Hypoxia is linked with a low pH and an acidic extracellular matrix microenvironment to genetic cell instability [4, 14, 110]. Mutation rates increase at lower pH with chromosomal breaks, chromosome gaps, chromosome exchanges (translocations), DNA over replication and gene amplification, gene expression changes, and lower cell survival [14, 110]. Cancers further resist chemotherapy and radiotherapy with lower pH acidic tissues in the tumor and the extracellular matrix [14, 110]. In addition, the cancer cell is neutral or slightly basic, whereas the extracellular matrix is acidic that prevents the use of weak base drugs [14, 110]. The cancer cell membrane excludes weak base drugs from entering the cell, allows nonpolar neutral drugs to enter, and increases permeability to weakly acidic drugs [14, 110]. To counteract acids for weak base drug delivery, the pH gradient at the cancer cell membrane can be eliminated in some mouse animal models with sodium bicarbonate ad libitum and through peritoneal injections [14, 110]. Other agents known to induce metabolic alkalosis in humans have further been recommended for adjunct chemotherapy to overcome hypoxic acidity in the tumor microenvironment [110].

## 11. Summary

Hypoxia initiates mitochondrial free radicals formed through the electron transport chain in addition to acid from the proton gradient. Multiple types of initiating events that interfere with oxygen to the mitochondria exist as cancer causing agents/environments. Free radicals are delocalized from the mitochondria for electron-pair bonding of proteins and initiate reactive secondary sequence chain growth of lipids with C=C double bonds to form structure at the molecular and cellular levels. Subsequent covalent crosslinking by electron-pair bonds can block oxygen to the mitochondria in an ever increasing vicious cycle of chronic free-radical production that can expand to the tissue level. Irregular cancer cell membrane borders are the result of free-radical lipid crosslink shrinkage and warpage that wrinkle and pucker the membranes inward when electron pairs bond. As membrane lipid chains come much closer together by electron-pair covalent-bond crosslinking and fit better onto the underlying cytoskeleton, structural actin fibers might also crosslink and wrinkle by shrinking inward. Hypoxic conditions that produce mitochondrial waste as acid and free radicals activate latent globular proteins into efficient molecular degrading enzymes. Enzymatic degradation is further modified by free radicals delocalized into globular proteins to buffer lower pH conditions formed by mitochondrial acids. Even when cancer cells switch to anaerobic glycolysis, high levels of lactate acid form to be easily measured in the extracellular matrix. Degradation by enzymes includes manifestations of multiple mutations in addition to epigenetic changes most notable involving carbon-carbon lipid bond dissociations to methylate DNA and silence tumor suppressor genes. Although DNA mutations, low pH enzyme activity, and free radical crosslinking drive most cells toward senescence and apoptosis, some cells survive through increasing levels of more incompatible environments to eventually produce immortal cancer cells through a genetic EMT. With EMT increasing toward more primitive cancer cell types, mitochondrial hypoxia and increased production from the electron transport chain and proton gradient are subsequent driving forces for cancer cell growth rather than cell death. Also, even when anaerobic glycolysis switches over, free radicals still form at high levels along with lactate acid to lower the surrounding pH for the extracellular matrix. Cancer proliferating cells have lower modulus than normal cells; whereby more cell growth occurs within the nucleus. Cells with lower modulus deform easier to invade local tissue and metastasize. Tissue invasion is increased by protease enzymes activated by hypoxic mitochondrial acid and free radicals. Metastasis finally occurs with more protease enzymes and high modulus membrane lamellipodia extensions that provide leverage and allow low modulus cells to deform through narrow openings in the blood vessel basement membrane. Lamellipodia growth increases with free radicals formed under hypoxic conditions through H_2_O_2_-elevated polymerization of actin fibers. Further, small membrane filipodia extensions especially off the lamellipodia protrusions provide focal adhesion points to pull cancer cells forward by contracting with the extracellular matrix as minor stronger primary covalent and much weaker secondary bonds form in a manner somewhat similar to cure-shrinkage contraction that occurs during covalent bond polymerization. Angiogenesis responsiveness to hypoxic tumor environment allows cells to survive. Cancer risks associated with obesity and caloric intake especially with dietary fats can now be better defined through risk factors involved in atherosclerosis of blood vessels that cause hypoxia and ischemia to tissue. Resultant decreased oxygen during cell metabolism produce mitochondrial free radicals and acid that are then directly involved in cancer. Other risk factors for cancer should also be defined in terms of possible ischemia and hypoxia at the mitochondrial level. The microenvironment surrounding the cancer tumor can eventually become hostile with free radicals and acid to an extent that macrophage killing is subverted to TAM tumors that might be better controlled toward health with free-radical inhibitors/antioxidants and various buffers. Finally, by acknowledging the original sources of pathology that include hypoxic mitochondrial free radicals and acid, corrective and preventive measures to remove or offset the initiating factors could be more easily done.

## Abbreviations

C=C: Carbon-carbon double bond
sigma (*σ*): Single bond
pi (*π*): Double bond
EMT: Epithelial-mesenchymal transition
TAM: Tumor-associated macrophage.
